# Pediatric trans-olecranon fracture dislocation of the elbow associated with fracture of the radial head and with medial collateral ligament lesion: A case report and review of the literature

**DOI:** 10.1016/j.tcr.2021.100399

**Published:** 2021-02-09

**Authors:** Daniele Massetti, Mario Marinelli, Rosa Palmisani, Valentino Coppa, Antonio Pompilio Gigante

**Affiliations:** a Department of Clinical and Molecular Science, Clinical Orthopedics, School of Medicine, Università Politecnica delle Marche, Ancona, Italy; b Clinic of Adult and Paediatric Orthopaedic, Azienda Ospedaliero-Universitaria, Ospedali Riuniti di Ancona, Ancona, Italy

**Keywords:** *Trans*-olecranon fracture dislocation, Pediatric elbow injury, Pediatric trauma, S.E.R.I. technique

## Abstract

**Introduction:**

We report what is, to the best of our knowledge, the first case of pediatric trans-olecranon fracture dislocation of the elbow associated with a radial head fracture and with a medial collateral ligament disruption.

**Case presentation:**

A 7-year-old girl presented to the emergency department after a fell on his right elbow while playful activity at home. The elbow X-ray showed acute trans-olecranon fracture dislocation of the elbow associated with a radial head fracture. A pre-operative 3D TC scans confirmed and clarified the injury pattern. However, stress radiographs performed in the operating room under anesthesia revealed an associated severe valgus instability caused by medial collateral ligament disruption. The olecranon fracture was fixed with two crossing 1.5 mm K-wires and the angulated radial neck fracture was fixed with a retrograde 1.5 mm K-wire by S.E.R.I. technique.

**Conclusion:**

Although trans-olecranon fracture dislocation of the elbow is well recognized and clearly described in adults, it is uncommon in children. A pre-operative 3D TC scans are recommended to enable a more accurate diagnosis and surgical planning. Medial collateral ligament has a central role in elbow stability and is very important to repair it during surgery.

## Introduction

Traumatic dislocation of the elbow is rare in children with an incidence of only 3% to 6% of all elbow injuries [1] and X-ray must be evaluated carefully to exclude associated injuries, like fractures or avulsions. We report what is, to the best of our knowledge, the first case of pediatric trans-olecranon fracture dislocation of the elbow associated with a radial head fracture and with a medial collateral ligament disruption who was surgically treated. *Trans*-olecranon fracture dislocation of the elbow can be defined a complex elbow instability in accord with Rotini classification [2]. The term complex elbow instability means the association of ligaments (Medial Collateral Ligament and/or Lateral Collateral Ligament) and bony lesions (usually the radial head and/or coronoid and/or olecranon process). The trans-olecranon fracture is characterized by the dislocation of the ulno-humeral joint caused by fracture of the olecranon process combined with injury of ligamentous stabilizers (Medial Collateral Ligament in this case). The trans-olecranon fracture dislocation is different from the anterior Monteggia (Bado 1) lesion because in the former, there is a loss of stability in the ulno-humeral joint, but the radioulnar relationship is preserved [3].

## Case presentation

**Fig. 1:**
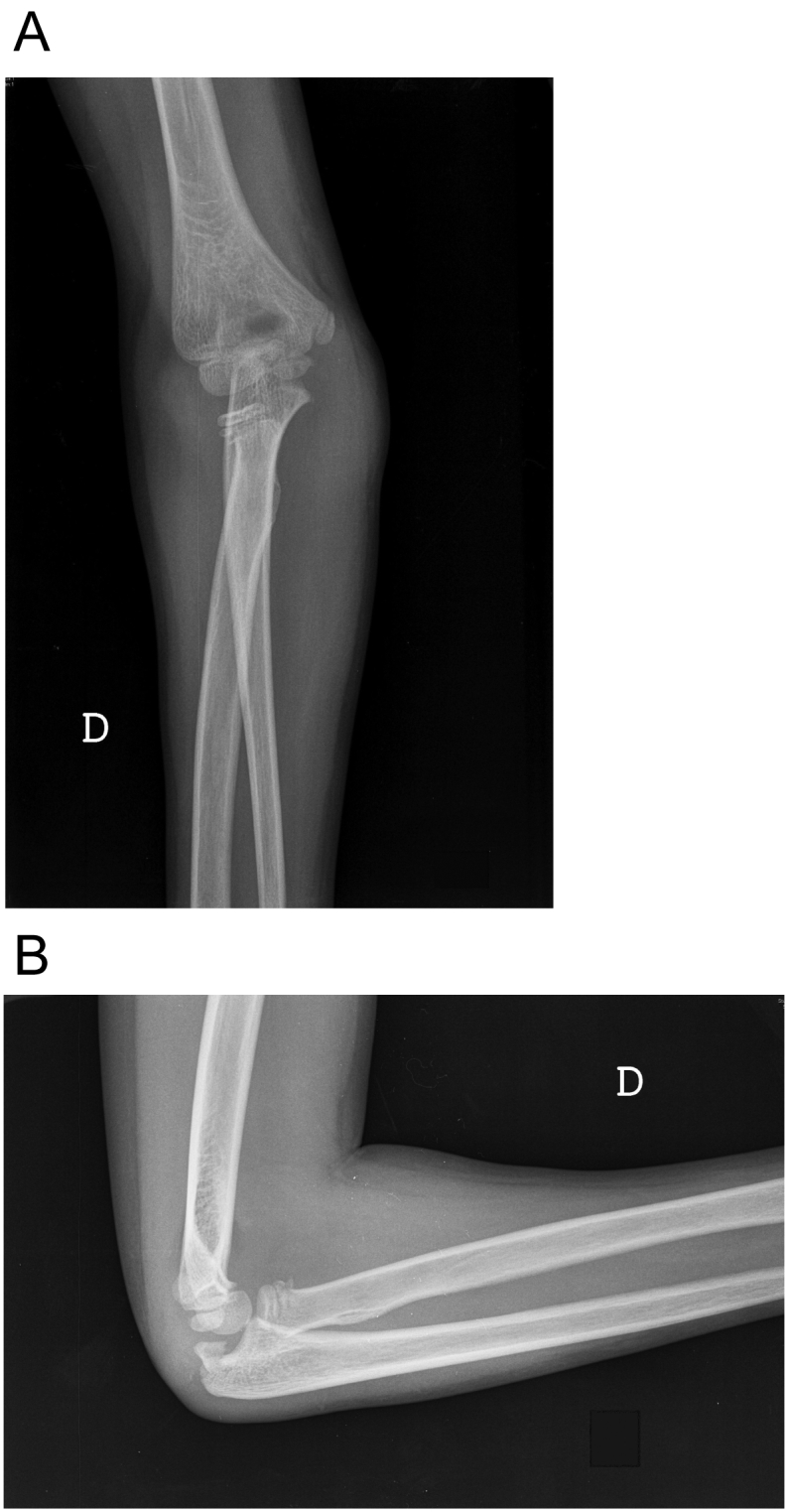
a–b: Standard elbow X-ray showed acute trans-olecranon fracture dislocation of the elbow associated with a radial head fracture.

**Fig. 2:**
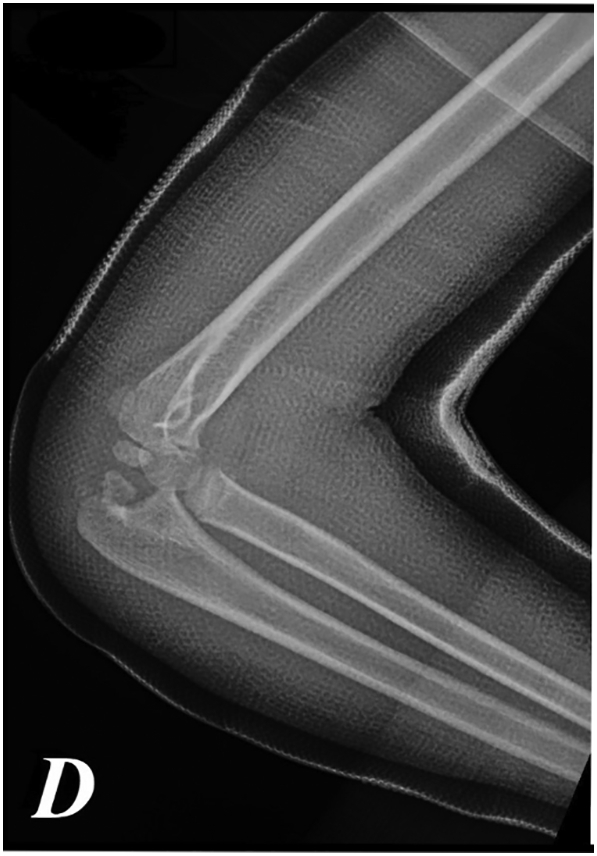
Elbow X-ray after a closed reduction attempt.

**Fig. 3:**
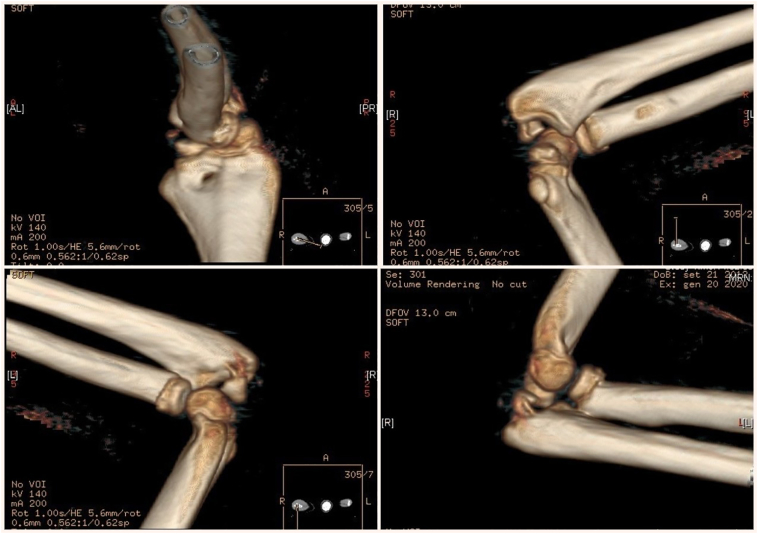
Pre-operative CT – scan with 3D reconstruction confirms an clarifies trans-olecranon fracture dislocation of the elbow associated with a radial head fracture.

**Fig. 4:**
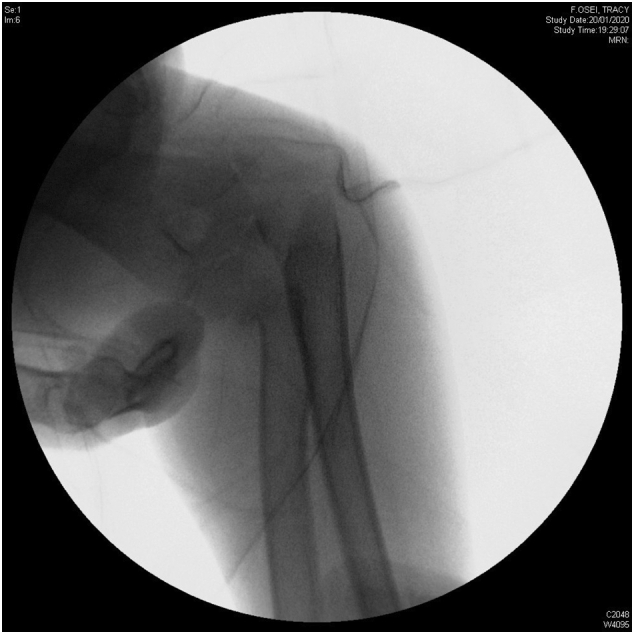
Stress radiographs performed in the operating room revealed an associated severe valgus instability caused by medial collateral ligament disruption.

A 7-year-old girl presented to the emergency department after a fell on his right elbow while playful activity at home. During physical examination, the patients' elbow range of motion was painful and limited. Their upper extremities were neurovascularly intact. The elbow X-ray showed acute trans-olecranon fracture dislocation of the elbow associated with a radial head fracture (Fig. 1a–b). Closed reduction under conscious sedation was attempted in the emergency department and the elbow was splinted in an above elbow plaster slab (Fig. 2). An urgent pre-operative 3D TC was performed that confirmed and clarified the injury pattern (Fig. 3). After the child was transferred to the operating room. However, stress radiographs performed in the operating room under anesthesia revealed an associated severe valgus instability caused by medial collateral ligament disruption (Fig. 4).

### Surgical technique

**Fig. 5:**
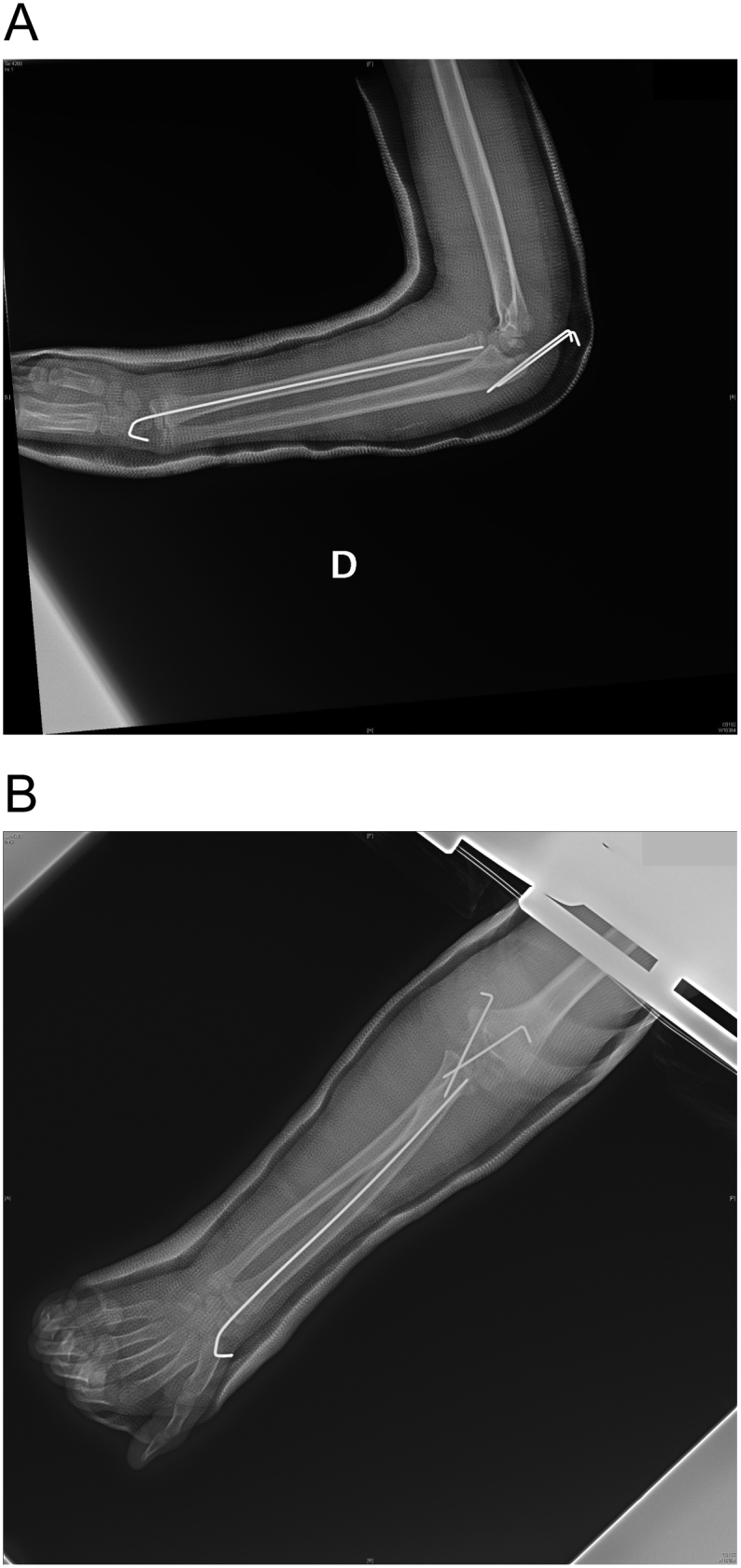
a–b: The post-operative elbow X-ray shows the olecranon fractures fixed with two crossing 1.5 mm K-wires and the angulated radial neck fracture fixed with a retrograde 1.5 mm K-wire by S.E.R.I. technique.

The patient is placed in the supine position, with shoulder at edge of bed, the upper limb affected at 90° on the chest placed over a support. The surgery was performed under intravenous sedation, spontaneous breathing and ultrasound guided brachial plexus block using 0.50% ropivacaine. Sedation drugs used were midazolam, fentanyl in association with ketamine and propofol. The local anesthetic used was ropivacaine 0.50%. A nonsterile pneumatic tourniquet is placed on the affected arm, then a sterile field is set starting from the proximal part of the upper limb down to the hand with the arm draped free. After an exsanguination of the arm performed by a sterile Esmarch bandage the pneumatic tourniquet is inflated. A posterior midline longitudinal incision is made to expose the olecranon and medial epicondyle. The ulnar nerve is identified and protected with retractor. During the ulnar nerve identification, we found that medial collateral ligaments were completely disrupted from the point of insertion to the distal humerus. The olecranon fractures were reduced and fixed with two crossing bicortical 1.5 mm K-wires. The radial neck fracture reduction was achieved with percutaneous 2.0 mm lanceolate K-wire leverage and definitively fixed with one 1.5 mm K-wire inserted percutaneously and dorsally in the wrist in accord with S.E.R.I. technique [4]. Medial collateral ligaments were attached to the distal humerus with a trans osseous absorbable suture fixation (Fig. 5a–b).

### Aftercare

Postoperatively, patients were immobilized with an above elbow cast at 90° flexion of the elbow with the forearm in neutral rotation for 4 weeks. A first X-ray was made 1 week after surgery. A second X-ray was performed after 4 weeks from surgery and after removing the cast and K-wire. Olecranon and radial neck K-wires were removed in the clinic without sedation or anesthesia and after the patients is allowed to return to daily activity avoiding contact sports for at least 4 weeks.

## Discussion

*Trans*-olecranon fracture-dislocation is well recognized and clearly described in adults [5], but it is uncommon in children. Only eleven cases of pediatric trans-olecranon fracture have been reported in literature [6] and so the management of this injury pattern is not clear. H. Tiemdjo et al. in 2015 propose a classification into four types of anterior trans-olecranon fracture-dislocations of the elbow in children, thereby guiding surgical indications [6]. Traditionally the management of this fracture has been surgical with tension band construct in avulsions and transverse fractures [7,8] and bone plate in oblique fractures in older children. Some Authors reported a nonsurgical management of trans-olecranon fracture dislocation in children with good results [9]. We fixed the olecranon fracture with two crossing bicortical 1.5 mm K-wires because in our opinion a k-wire fixation is sufficient for treating a pediatric olecranon fracture avulsion especially in very young children. Furthermore, this olecranon fixation allows to remove K-wires in the clinic without sedation or anesthesia after 4 weeks. The fixation with tension band construct requires a second surgery for remove the implants. Medial collateral ligament, especially the anterior band, has a central role in elbow stability, in particular against valgus instability [10]. The anterior band of medial collateral ligament acts as the major stabilizing ligamentous structure in the elbow and it is divided into two functional components, which allows it to remain taut throughout the full range of flexion and extension. For this reason, it's very important to repair medial collateral ligament during surgery with a trans osseous absorbable suture or with a suture anchor.

## Conclusion

*Trans*-olecranon fracture dislocation of the elbow associated with a radial head fracture and with a medial collateral ligament disruption is a rare subgroup of complex elbow instability. This injury pattern can be challenging to diagnose because large portion of the elbow is radiolucent in young children. A pre-operative 3D TC scans are recommended to enable a more accurate diagnosis and surgical planning. Medial collateral ligament has a central role in elbow stability and is very important to repair it during surgery.

## Declaration of competing interest

None.
